# Annual acknowledgement of manuscript reviewers

**DOI:** 10.1186/1471-2156-14-15

**Published:** 2013-03-20

**Authors:** Simon Harold

**Affiliations:** 1 BioMed Central, Floor 6, 236 Gray's Inn Road, London, WC1X 8HB, United Kingdom

## Abstract

**Contributing reviewers:**

The editors of *BMC Genetics* would like to thank all of our reviewers who have contributed to the journal in Volume 13 (2012).

## 

Laurent Abi-Rached

France

Cheryl Ackert-Bicknell

USA

Barnaud Adeline

France

Ilir Agalliu

USA

Karen Aitken

Australia

Katharina Viktoria Alheit

Germany

Roberto Amato

Italy

Leif Andersson

Sweden

Maria Grazia Andreassi

Italy

Razvan Anistoroaei

Denmark

Ian Armstead

UK

Hugues Aschard

USA

Yurii Aulchenko

Russian Federation

Philip Awadalla

Canada

Awoyemi Awofala

Nigeria

Bojlul Bahar

Ireland

Niranjan Baisakh

USA

Kristian Baker

USA

Juan Barcena

Spain

Noelle Barkley

USA

Stefan Bekiranov

USA

Elisa Bellucci

Italy

Olga Belyaeva

USA

Elena Benavente

Spain

Cesar Benito

Spain

Donagh Berry

Ireland

Luiz Bertollo

Brazil

Elena Bitocchi

Italy

Antonio Blanco

Italy

Andrea Blas

Spain

Folmer Bokma

Sweden

Udayan Borthakur

India

Henk Bovenhuis

Netherlands

Peter Bradbury

USA

Dan Bradley

Ireland

Martin Braunschweig

Switzerland

Karl Broman

USA

Reto Burri

Sweden

Erkan Buzbas

USA

Graciela Cabana

USA

Diogo Cabral-de-Mello

Brazil

Juan Pedro Camacho

Spain

Daniel Camerini-Otero

USA

Nicola Camp

USA

Maximiliano Canepa

Australia

Moju Cao

China

Luis Carvajal Carmona

UK

Gary Carvalho

UK

Eduardo Casas

USA

Richard Cawthon

USA

Swarup Chakrabarti

India

Dalia Chakrabarty

UK

Deborah Charlesworth

UK

Jean-Eric Chauvin

France

Charles Chen

USA

Zi-Jiang Chen

China

Lin Chen

USA

Hans Cheng

USA

Mete Civelek

USA

Don Colgan

Australia

Jordi Comadran

UK

Heather Cordell

UK

Fabiane Costa

Brazil

A. Morrie Craig

USA

David Crockett

USA

Kevin Crosby

USA

John J. Crowley

Ireland

Phat Dang

USA

Roy Danzmann

Canada

Catherine Dargemont

France

Kiranmoy Das

USA

Steve Davis

New Zealand

Roberta Davoli

Italy

Bernard de Massy

France

Tim De Meyer

Belgium

Santie De Villiers

Kenya

Ruud Delwel

Netherlands

Olivier Demeure

France

Gabriele Di Gaspero

Italy

Xiangdong Ding

China

Assaf Distelfeld

Israel

Narendra Dixit

India

Lu Dong

China

Gaby Doxiadis

Netherlands

Zhi-Qiang Du

USA

Canxing Duan

China

Lisa Durso

USA

Julien Dutheil

France

Scott Edwards

USA

Jean Michel Elsen

France

Livinus Emebiri

Australia

Scott Emrich

USA

Jürg Enkerli

Switzerland

Warren Ewens

USA

João Fadista

Denmark

Patricia Faivre Rampant

France

Rui Feng

USA

Willem Ferguson

South Africa

Juan Jose Ferreira

Spain

Iris Fischer

France

Pierre Fobert

Canada

Marie Fogarty

USA

Luca Fontanesi

Italy

Jia Nee Foo

Singapore

Jiri Forejt

Czech Republic

Selma Forni

USA

Alexandre Fournier-Level

Australia

Paolo Franchini

Germany

Merete Fredholm

Denmark

Gertraude Freyer

Germany

Donald Frohlich

USA

Agata Gadaleta

Italy

Guimin Gao

USA

Amber Garber

Canada

Maria J Garcia-Garcia

USA

Dorian Garrick

USA

Carine Genet

France

Nicolas Gengler

Belgium

Chris Gignoux

USA

Bikram Gill

USA

Damjan Glavac

Slovenia

Tom Goldammer

Germany

Luis Gomez-Raya

USA

Elena G. Gonzalez

Spain

Jan Graffelman

Spain

Alexander Graphodatsky

Russian Federation

Michael Grashorn

Germany

Thomas Gridley

USA

Yongtao Guan

USA

Michele Guescini

Italy

Baozhu Guo

USA

René Guyomard

France

Chris Haley

UK

Leng Han

USA

Olivier Hanotte

UK

David Heckel

Germany

John Henshall

Australia

Pilar Hernandez

Spain

Bruce Herron

USA

Nicolas Heslot

USA

John Hickey

Australia

William Hill

UK

Axel Hillmer

Singapore

Suzanne Hizer

USA

Paul Hocking

UK

Ola Hössjer

Sweden

Ross Houston

UK

Ting Hu

USA

Wen Huang

USA

Xin Huang

USA

Eveline Ibeagha-Awemu

Canada

Akihiro Ikeda

USA

Wilfred Ip

Canada

Moritoshi Iwagami

Japan

Naoharu Iwai

Japan

Mandy Jackson

UK

Hans-Georg Joost

Germany

Jordi Jordana

Spain

Ki-Hong Jung

South Korea

Kazusaku Kamiya

Japan

Raj Kandpal

USA

Chen-Hung Kao

Taiwan

Manfred Kayser

Netherlands

Matthew Kelly

Australia

Brian Kemp

USA

Peter Kerr

Australia

Mehar Khatkar

Australia

Kenneth K. Kidd

USA

Masato Kinoshita

Japan

Brian Kirkpatrick

USA

Roland Kölliker

Switzerland

Lukas Kratochvil

Czech Republic

Johannes Krause

Germany

Christa Kuehn

Germany

Gustavo Kuhn

Brazil

Mary Kuhner

USA

Poh San Lai

Singapore

Carles Lalueza-Fox

Spain

Matthew Lanktree

Canada

Oscar Lao

Netherlands

Luisa Last

Switzerland

Uri Lavi

Israel

Young Sik Lee

South Korea

Andres Legarra

France

Sigrid Lehnert

Australia

Yao Li

USA

Yun Li

USA

Xi-Xiang Li

China

Zitong Li

Finland

Hongying Li

USA

Shaoyu Li

USA

Ming Li

USA

Jiahan Li

USA

Gengxin Li

USA

Zhaohai Li

USA

Eric Liebgold

USA

Honghuang Lin

USA

Wan-Yu Lin

USA

Anna Linderholm

UK

Bang Liu

China

Jian-Feng Liu

China

Jingyuan Liu

USA

Sunny Liu

USA

Douglas Londono

USA

Christian Looft

Germany

Marta Lopes

Turkey

Luciana Lourenço

Brazil

James Luby

USA

Adam Lukaszewski

USA

Anne Lundén

Sweden

David Lynn

Ireland

Wujun Ma

Australia

Nicolo Macciotta

Italy

Margaret Mackinnon

Kenya

Stuart MacNeill

UK

Gregory Maes

Belgium

Clint Magill

USA

Thibaut Malausa

France

Christian Maltecca

USA

Mauro Mandrioli

Italy

Douglas Marchuk

USA

Daniela Marone

Italy

Cesar Martins

Brazil

Anna Maria Mastrangelo

Italy

Hisao Masukata

Japan

Raluca Mateescu

USA

Scot Matkovich

USA

Celia May

UK

Bruce McDonald

Switzerland

Jan McDowell

USA

John McEwan

New Zealand

Tim McNellis

USA

Kieran Meade

Ireland

Hendrik-Jan Megens

Netherlands

Emese Meglecz

France

Eli Meyer

USA

Thomas Miedaner

Germany

Stephen Miller

Canada

Thomas Moen

Norway

Seyed Abolghasem Mohammadi

Iran

Marta Molnar-Lang

India

Stefano Mona

France

Samrat Mondol

India

Xavier Montagutelli

France

Richard Moore

USA

Christopher Moran

Australia

Gerhard Moser

Australia

Connie Mulligan

USA

Indrajit Nanda

Germany

Alexander Nater

Switzerland

Alejandro Nato

USA

Alireza Navabi

Canada

Dana Nelson

USA

Matthew Nelson

Australia

Frank Nicholas

Australia

Wen Hui Nie

China

Anita Oberbauer

USA

Dennis O'Brien

USA

Konrad Ocalewicz

Poland

Nathan O'Callaghan

Australia

Damaris Odeny

South Africa

Devin Oglesbee

USA

Kevin Ohlemiller

USA

Ettore Olmo

Italy

Rafael Oriol

France

Elaine A Ostrander

USA

Antonio Pacheco

Mexico

Per Palsboll

Netherlands

Pekka Pamilo

Finland

Wei Pan

USA

Roberto Papa

Italy

Heidi Parker

USA

Francisco Peñagaricano

USA

Bo Peng

USA

Andy Pereira

USA

Miguel Perez-Enciso

Spain

Silvina Pessino

Argentina

Gary Peter

USA

Hans-Peter Piepho

Germany

Marie-Hélène Pinard-van der Laan

France

Matti Pirinen

UK

Geoffrey Pollott

UK

Francois Pompanon

France

Elena Potokina

Russian Federation

Paul Proost

Belgium

Catherine Purcell

USA

Herman Raadsma

Australia

Harsh Raman

Australia

Rosy Raman

Australia

Nirala Ramchiary

South Korea

Kenneth Ramos

USA

Domenico Rau

Italy

Terje Raudsepp

USA

Carol Reeb

USA

Danielle Reed

USA

Giuseppe Reforgiato Recupero

Italy

Birgitte Regenberg

Denmark

Jose A. Riancho

Spain

Juliette Riquet

France

Marion Roder

Germany

Monica Rodriguez

Italy

Odd Arne Rognli

Norway

Gary Rohrer

USA

Megan Rolf

USA

Mikeal Roose

USA

Janet Rossant

USA

Josep A. Rossello

Spain

Mathieu Rougemaille

France

Carl-Johan Rubin

Sweden

Peter Ryan

Australia

Alexei Ryskov

Russian Federation

Goutam Sahana

Denmark

Antonio Sanchez

Spain

Raquel Sánchez-Pérez

Spain

Jessica Satkoski Trask

USA

Ulrike Sauermann

Germany

Richa Saxena

USA

Robert Schnabel

USA

James Schneider

USA

Colin Semple

UK

Y.D. Sharma

India

Vinay Shenoy

India

Peng Shi

China

Sukhwinder Singh

Mexico

Montgomery Slatkin

USA

Warren Snelling

USA

Minsun Song

USA

Tad Sonstegard

USA

Vitor Sousa

USA

Peerasak Srinives

Thailand

Juan Steibel

USA

Kathrin Friederike Stock

Germany

Anne Stone

USA

Paul Stothard

Canada

Ismo Stranden

Finland

Hokeun Sun

USA

Nathan Sutter

USA

Carolyn Sweeney

USA

Marek Switonski

Poland

E Shyong Tai

USA

Akshay Talukdar

India

Sheh May Tam

Malaysia

Eduardo Tarazona-Santos

Brazil

Diethard Tautz

Germany

Ross Tellam

Australia

Yik Ying Teo

Singapore

Raffaele Testolin

Italy

William Thomas

UK

Ralph Tiedemann

Germany

Fausto Tinti

Italy

Vladimir Trifonov

Russian Federation

David Tritchler

Canada

Tudorita Tumbar

USA

Benjamin Tycko

USA

V Udhayakumar

USA

George Uhl

USA

Ales Vancura

USA

Marco Vanoni

Italy

Paul VanRaden

USA

Edwin Veldhuizen

Netherlands

Ignazio Verde

Italy

Fabio Veronesi

Italy

Marcelo Vicari

Brazil

Alain Vignal

France

Kunjupillai Vijayan

India

Johanna Vilkki

Finland

Vitaly Volobouev

France

Daniel Wagner

USA

Tao Wang

USA

Xiaofeng Wang

USA

Xuexia Wang

USA

Zuoheng Wang

USA

Xuefeng Wang

USA

Chenguang Wang

USA

Robin Waples

USA

Craig Warden

USA

Paul Waters

Australia

Scott Weatherbee

USA

Lee Weigt

USA

George Wiggans

USA

James Wilson

UK

Steve Wilton

Australia

Klaus Wimmers

Germany

Melodie Winawer

USA

Jason Wolf

UK

Song Wu

USA

Shu-Biao Wu

Australia

Michael Wu

USA

cen wu

USA

Junhua Xiao

China

Lizhong Xiong

China

Peng Xu

China

Shizhong Xu

USA

Li Xu

USA

Rattan Yadav

UK

Xiting Yan

USA

Wenzhao Yang

USA

Glenn Yannic

Switzerland

Weichuan Yu

Hong Kong

Zhaoxia Yu

USA

Jing Zhang

USA

Wei Zhao

USA

Xin Zhao

Canada

Degui Zhi

USA

Shujun Zhou

China

Xiaofeng Zhu

USA

Xu-xiao Zong

China

Fei Zou

USA

